# Supplementary material for: Comprehensive Annotation and Functional Exploration of MicroRNAs in Lettuce
Source: Front Plant Sci. 2021 Dec 24;12:781836. doi: 10.3389/fpls.2021.781836 (PMC8739914; doi:10.3389/fpls.2021.781836)
Supplement: Supplementary file 2 [file Data_Sheet_8.ZIP › GSM2230749.plot/Lsa-miR156i_Lsat_1_v5_gn_4_81541.1_850_TPlot.pdf]

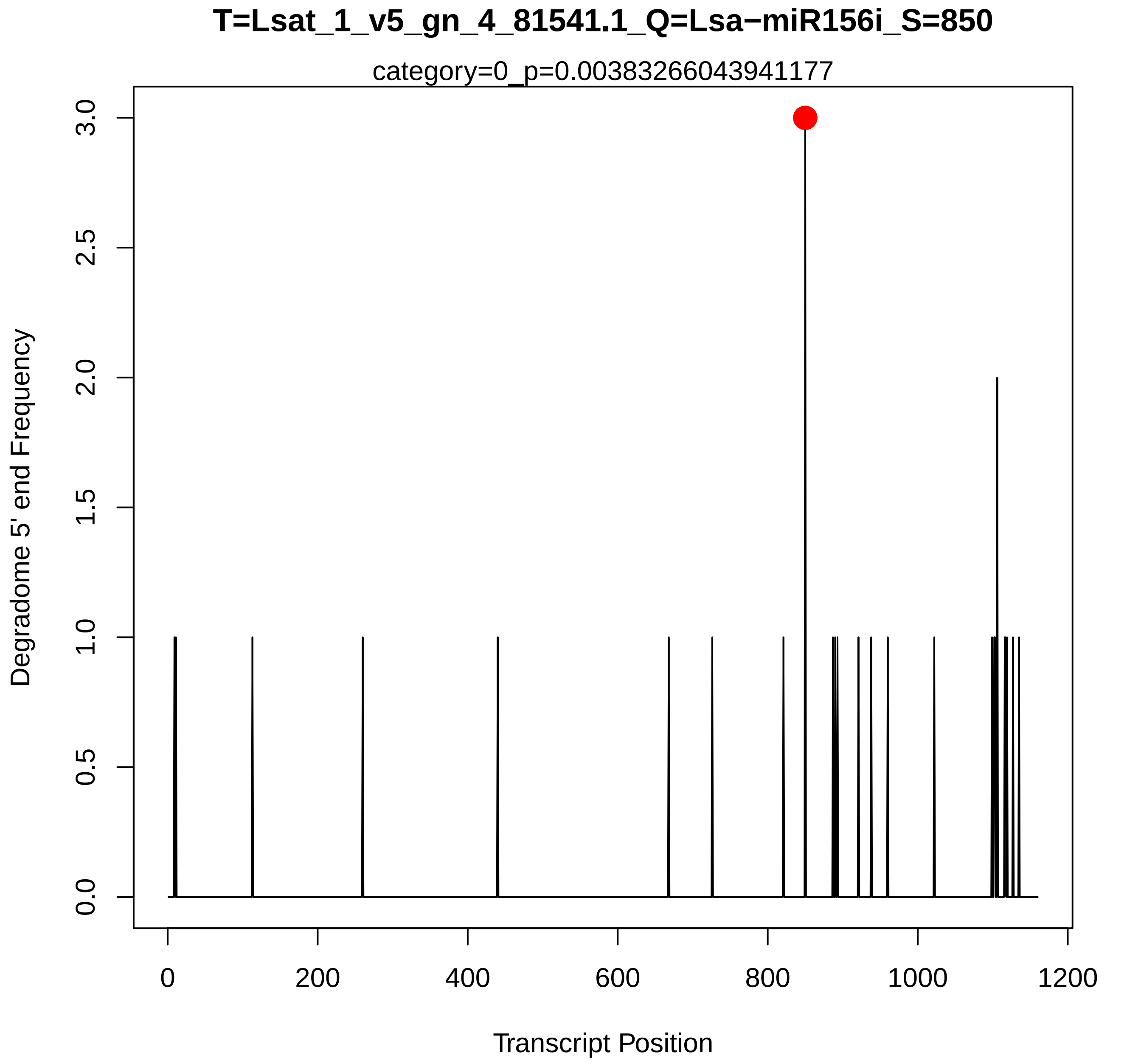

T=Lsat_1_v5_gn_4_81541.1_Q=Lsa−miR156i_S=850
category=0_p=0.00383266043941177
Degradome 5' end Frequency
0.0
0.5
1.0
1.5
2.0
2.5
3.0
0
200
400
600
800
1000
1200
Transcript Position
